# Oscillatory Deficits in the Sub-Chronic PCP Rat Model for Schizophrenia Are Reversed by mGlu5 Receptor-Positive Allosteric Modulators VU0409551 and VU0360172

**DOI:** 10.3390/cells12060919

**Published:** 2023-03-16

**Authors:** Jessica Brown, Ben Grayson, Joanna C. Neill, Michael Harte, Mark J. Wall, Richard T. Ngomba

**Affiliations:** 1 Division of Pharmacy & Optometry, University of Manchester, Manchester M13 9PT, UK; 2 School of Life Sciences, University of Warwick, Coventry CV4 7AL, UK; 3 School of Pharmacy, University of Lincoln, Lincoln LN6 7DL, UK

**Keywords:** metabotropic glutamate receptor, allosteric modulators, intracellular signalling, VU0409551, VU0360172, gamma oscillations, schizophrenia, phencyclidine (PCP), cognition, rat

## Abstract

The cognitive deficits of schizophrenia are linked to imbalanced excitatory and inhibitory signalling in the prefrontal cortex (PFC), disrupting gamma oscillations. We previously demonstrated that two mGlu5 receptor-positive allosteric modulators (PAMs), VU0409551 and VU0360172, restore cognitive deficits in the sub-chronic PCP (scPCP) rodent model for schizophrenia via distinct changes in PFC intracellular signalling molecules. Here, we have assessed ex vivo gamma oscillatory activity in PFC slices from scPCP rats and investigated the effects of VU0409551 and VU0360172 upon oscillatory power. mGlu5 receptor, protein kinase C (PKC), and phospholipase C (PLC) inhibition were also used to examine ‘modulation bias’ in PAM activity. The amplitude and area power of gamma oscillations were significantly diminished in the scPCP model. Slice incubation with either VU0409551 or VU0360172 rescued scPCP-induced oscillatory deficits in a concentration-dependent manner. MTEP blocked the PAM-induced restoration of oscillatory power, confirming the requirement of mGlu5 receptor modulation. Whilst PLC inhibition prevented the power increase mediated by both PAMs, PKC inhibition diminished the effects of VU0360172 but not VU0409551. This aligns with previous reports that VU0409551 exhibits preferential activation of the phosphatidylinositol-3-kinase (PI3K) signalling pathway over the PKC cascade. Restoration of the excitatory/inhibitory signalling balance and gamma oscillations may therefore underlie the mGluR5 PAM-mediated correction of scPCP-induced cognitive deficits.

## 1. Introduction

Schizophrenia is a severe psychotic condition characterised by positive (delusions, visual and auditory hallucinations,) and negative (social withdrawal, flat affect) symptom clusters [1]. In addition, impaired cognitive functions (including working memory and cognitive flexibility) are increasingly recognised as a core clinical aspect of the disorder. The cognitive impairment associated with schizophrenia (CIAS) is remarkably robust, associated with the initial, prodromal stages of schizophrenia and closely linked to functional outcomes and quality of life [2,3,4,5,6,7]. A recent cohort study revealed that the start of cognitive decline precedes psychosis onset by over a decade, implying that cognitive deficits represent the underlying pathophysiology of schizophrenia, with psychosis being a secondary symptom [8]. Existing typical and atypical antipsychotic medications modifying monoamine receptors have shown value in mitigating the positive, but neither negative nor cognitive, symptoms [9]. Moreover, the major side effects elicited by currently available treatments, ranging from cardiometabolic disturbances to motor dysfunction, mean that the majority (74%) of patients discontinue their medication within 18 months [10]. Thus, there is urgent pressure to develop safe, selective compounds acting at novel targets to improve CIAS.

Current research is moving away from the dopaminergic hypothesis of schizophrenia, and there is now mounting evidence that glutamatergic aberrations may contribute to those symptoms that are resistant to antipsychotic medications [11,12]. A subset of interneurons in the prefrontal cortex (PFC) express parvalbumin (PV), an activity-dependent calcium-binding protein often showing reduced expression in pathological states [13]. These PV basket interneurons play an integral role in cognition, delivering GABAergic inhibitory signals to pyramidal cell perisomas, which in turn mediate glutamatergic excitation [14]. Notably, the equilibrium between levels of inhibition and excitation in the PFC is not static. By contrast, the plasticity of this excitatory/inhibitory interplay fosters long-term potentiation (LTP) and the flexibility of PFC activity [15,16]. An abundance of research draws associations between CIAS and the disruption of this PFC microcircuit [17]. Specifically, cognitive deficits are attributed to the reduced GABAergic inhibition of pyramidal neurons by PV interneurons, which consequently disrupts the excitatory/inhibitory balance. Indeed, GAD67 mRNA could only be measured in 55% of PV mRNA-positive neurons in schizophrenia subjects, with patients also showing significantly reduced PV mRNA expression in the PFC [18]. In vivo data from rats also reveal bicuculline-mediated antagonism of PFC GABA-A receptors to instigate excitatory/inhibitory signalling disturbances and impair the key PFC-dependent cognitive abilities of working memory and cognitive flexibility [19,20]. Gamma oscillations are heavily reliant upon the precise balance between PFC pyramidal cells and inhibitory interneurons [21]. By controlling the integration within cortical circuits, oscillations provide the temporal coordination of neuronal activity necessary for functions such as memory, attention, perception, and synaptic plasticity. Accordingly, mounting evidence from preclinical and clinical studies suggests that oscillatory power in the gamma band is significantly diminished in schizophrenia [22,23,24].

In light of these data, selectively strengthening glutamatergic inputs to PFC PV interneurons is hypothesised to re-align the excitatory/inhibitory signalling balance, correct deficits in gamma oscillations, and alleviate cognitive dysfunction. Metabotropic glutamate (mGlu) receptors are promising candidate drug targets to achieve this, as reviewed by Nicoletti et al. [25]. These receptors impact glutamatergic transmission in major schizophrenia-associated brain regions and thus represent a novel avenue to develop superior pharmacotherapies for schizophrenia [26]. These G-protein-coupled receptors are categorised into three groups: Group I mGlu receptors typically couple to Gq/11 proteins and include mGlu1 and mGlu5 receptors; group II and group III couple to Gi/o proteins and include mGlu2 and mGlu3 receptors (group II) and mGlu4, mGlu6, mGlu7, and mGlu8 receptors (group III) [27]. In particular, the mGlu5 receptor is evidenced to play an integral role in the glutamatergic dysfunction of schizophrenia and is strategically positioned as a target to correct oscillatory deficits. Not only is this receptor present on GABAergic PV interneurons and functionally coupled to the NMDA receptor, but there is much evidence linking altered mGlu5 receptor functioning to the pathology of schizophrenia [28,29]. Positive allosteric modulators (PAMs) couple to an allosteric site on the mGlu5 receptor, enhancing the responsiveness of the receptor to glutamate without activating the receptor itself. The ability of PAMs to refine endogenous agonist activity confers advantages such as greater selectivity and reduced side effects [30]. mGlu5 receptor PAMs have been demonstrated to enhance cognitive performance, improve recognition memory, attenuate avoidance response, and reduce impulsivity in the five-choice serial reaction time task in rodents [31,32,33,34,35]. We have shown that the potent, selective mGlu5 receptor PAMs VU0409551 and VU0360172 significantly reverse cognitive deficits in the scPCP rat model for schizophrenia [36] and they are also efficacious in acute psychosis models [37,38]. 

mGlu5 receptor activation stimulates multiple independent signalling pathways. Briefly, mGlu5 receptor-activated Gαq/11 stimulates phospholipase C (PLC) to generate inositol-1,4,5-triphosphate (IP3), leading to intracellular calcium mobilisation [39]. Activated PLC also produces diacylglycerol (DAG), which in turn phosphorylates PKC, MAPK, and ERK and leads to downstream modulation of several ion channels [40,41,42]. mGlu5 receptor stimulation can alternatively activate the phospha-tidyl-inositol-3kinase (PI3K) pathway, resulting in the phosphorylation of AKT and activation of the mammalian target of rapamycin (mTOR) [43,44]. mGlu5 receptor PAMs may be able to modulate certain signalling pathways to achieve desirable therapeutic responses whilst avoiding the stimulation of others, termed ‘biased’ modulation [45]. An early study into this selectivity demonstrated that whilst both mGlu5 receptor PAMs DFB and CPPHA induced intracellular calcium release, they induced differential effects on ERK1/2 signalling [46]. We previously showed VU0409551 and VU0360172 to exhibit divergent effects on scPCP-induced increases in PFC AKT and MAPK phosphorylation, with VU0409551 inducing a significant alteration in expression of p-AKT, and VU0360172 modifying p-MAPK levels [36]. Other publications support the hypothesis that these PAMs differ in their modulation of mGlu5 receptor-linked signalling pathways: VU0409551 did not modulate pERK1/2 levels in the hippocampus or PFC when dosed chronically and was biased away from the PKC/pERK1/2 pathway relative to VU0360172 [35,47]. VU0409551 also does not potentiate the NMDA receptor, which may minimise excitotoxic side effects [38]. 

Here we have employed the sub-chronic PCP (scPCP) preclinical rat model for CIAS to investigate the effect of scPCP treatment on ex vivo gamma oscillations in the PFC and evaluated the impact of mGlu5 receptor PAMs VU0360172 and VU0409551 on oscillatory activity. We also manipulated downstream signalling components to explore any ‘biased’ modulation in the activity of these compounds. This is the first time these PAMs have been assessed in the scPCP model at a network level, which is a critical bridge in relating cellular mechanisms to complex behaviours. In this instance, the relationship between previously demonstrated improved novel object recognition (NOR) performance and changes in intracellular signalling molecules is investigated at a physiological level.

## 2. Materials and Methods

### 2.1. Animals

Overall, we have used 54 female Lister Hooded rats (Cohort 1, *n* = 25; Cohort 2, *n* = 29; Charles River Laboratories, UK) weighing an average of 207.3 g prior to study commencement, and these rats were group housed (4–5 per cage) in GR900 individually ventilated cages at 20–24 °C, 55 ± 10% humidity on a 12 h light/dark cycle (lights on at 0700h). Environment enrichment was supplied via paper wool and cardboard play tunnels (all IPS). Throughout the experiments, animals were allowed access to standard rodent diet pellets (Special Diet Services) and water ad libitum. Procedures were performed at the University of Warwick, approved by University of Warwick Animal Welfare and Ethical Review Board (AWERB), and were in compliance with the Home Office Animals (Scientific Procedures) Act 1986. scPCP dosing and behavioural testing at the University of Warwick was covered by secondary availability of the University of Manchester project license P763B36B8. 

### 2.2. Drugs

Phencyclidine (PCP; Sigma-Aldrich, Gillingham, UK; dissolved in 0.9% saline solution); VU0360172 (*N*-cyclobutyl-6-((3-fluorophenyl)ethynyl)picolin-amide; Tocris (Bio-Techne), Pittsburgh, USA; stock 10 mM in DMSO); VU0409551(4-fluorophenyl)(2-(phenoxymethyl)-6,7-dihydrooxazolo[5,4-*c*]pyridin-5(4*H*)-yl)methanone; Tocris (Bio-Techne), Pittsburgh, USA; stock 10 mM in DMSO); Kainic acid (Hello Bio, Bristol, UK; stock 0.25 mM, in dH_2_O); Carbachol (Carbamoylcholine chloride; Sigma-Aldrich, Gillingham, UK; stock 50 mM in dH_2_O); MTEP (3-((2-Methyl-4-thiazolyl)ethynyl)pyridine; Hello Bio, Bristol, UK; stock 200 µM in DMSO); Go6983 (Hello Bio, Bristol, UK; stock 10 µM in DMSO); U73122 (Abcam, Cambridge, UK; stock 10 mM in DMSO).

### 2.3. Drug Treatments

Animals were dosed with either sub-chronic vehicle (scVehicle, 0.9% saline; Cohort 1, *n* = 12) or scPCP (2 mg/kg; Cohort 1, *n* = 13; Cohort 2, *n* = 29) dissolved in 0.9% saline, via the intraperitoneal route (i.p.) twice daily for 7 days, followed by a 6-week washout period. A washout period of at least 1 week following scPCP dosing is required to avoid direct drug or drug withdrawal effects impacting animal behaviour [48]. The 6-week washout period used in the current study was informed by our previous findings that robust deficits in PV expression in the scPCP model are only established after a 6-week washout period [49,50]. In both Cohorts, animals were culled after NOR testing, and acute PFC slices were obtained for subsequent ex vivo electrophysiological experiments (methods outlined below).

### 2.4. Behaviour: Novel Object Recognition Paradigm

The NOR test was conducted on both animal Cohorts as previously explained [51,52]. Briefly, habituation of the empty test box (52 cm × 52 cm × 31 cm) and test room environment for 20 min was performed in cage groups 24 h before NOR testing. The paradigm comprised two 3 min trials divided by a 1 min inter-trial interval (ITI) in the home cage. In the first (acquisition) trial, each animal was presented with two identical objects in the test box. Subsequently, the second (retention trial) featured a duplicate (without olfactory trails) familiar object from the acquisition phase accompanied by a novel object. Randomisation of the positioning (left/right) and type (can/bottle) of object was performed to avoid the confounding effects of object and place preference. Video recordings of animal exploration were scored by a blinded experimenter using the ‘Jack R Auty Novel Object Recognition Task Timer’. Exploration of each object in each trial was specified as the rat licking, sniffing, or touching the object with the forepaws whilst sniffing, but behaviours such as leaning against, turning around, standing or sitting on the object were not counted as exploration [50]. Locomotor activity (LMA) across both trials was also assessed by the number of times the base of an animal’s tail crossed a marked line. The discrimination index (DI) was calculated as the difference in exploration time between objects expressed as a proportion of the total object exploration time. If an animal showed no exploration of one or both objects (for less than 1 s) in either trial, it was excluded from the data analysis.

### 2.5. Slice Preparation and Incubation

Coronal slices containing the infralimbic cortex (IL) were prepared as follows: rats (15–20 weeks old) were culled using CO_2_ overdose and then quickly decapitated. The brain was removed and immediately submerged in ice-cold (2–4 °C), high Mg^2+^, low Ca^2+^ artificial CSF (aCSF), composed of the following (in mM): 127 NaCl, 1.9 KCl, 8 MgCl_2_, 0.5 CaCl_2_, 1.2 KH_2_PO_4_, 26 NaHCO_3_, 10 D-glucose (pH 7.4 when bubbled with 95% O_2_/5% CO_2_). Slices (450 µm) of the PFC were cut between +1.7 mm and +3 mm with respect to Bregma coordinates [53] using the Microm HM 650V microslicer in aCSF. Slices were then moved to a holding chamber containing standard aCSF (1 mM MgCl_2_, 2 mM CaCl_2_) and continuously bubbled with 95% O_2_/5% CO_2_. Slices were left to recover at 30 °C for at least 1 h before recordings. For the recording of oscillations, slices were transferred to an interface-style recording chamber (Digitimer BSC3) and constantly perfused with warm (30 °C) aCSF at 6.67 mL/min. Slices from Cohort 1 were then incubated for 1 h with a solution of aCSF containing either the vehicle DMSO (control, 1:2000), VU0409551 dissolved in DMSO (2–5 µM) or VU0360172 dissolved in DMSO (2–5 µM) via bath application. In Cohort 2, these treatments were compared to slices co-incubated with the mGlu5 receptor PAMs alongside the mGlu5 receptor inhibitor MTEP (1 µM), the PKC inhibitor Go6983 (10 nM) or the PLC inhibitor U73122 (10 µM), with the inhibitor applied 20 min prior to each mGlu5 receptor PAM.

### 2.6. Induction of Oscillations Ex Vivo

Following the 1 h incubation with the PAMs (or vehicle), slices were washed with aCSF for at least 20 min before the oscillatory activity was induced. Extracellular field potential activity was recorded using a microelectrode filled with aCSF positioned in layer V of the IL cortex, identified using a brain atlas [53]. Oscillatory activity was elicited by bath application of Kainic acid (KA; 500 nM) and carbachol (50 µM) and recorded for at least 30 min. We chose this time period for recordings to maximise the number of recordings that could be made each day and reduce animal numbers required. Thus, our data reflect changes in the onset of oscillations rather than changes that may occur when they reach steady state after 2–3 h. Gamma oscillations were recorded with a differential amplifier (Warner instrument DP-301) with filter settings: low pass 3 KHz, high pass 1 Hz, amplification ×1000. Data were acquired at a sampling rate of 10 KHz with a Micro1401 (Cambridge Electronic Design) using Spike 2 software.

### 2.7. Analysis

#### 2.7.1. NOR Data

A two-way ANOVA with multiple comparisons (factors: drug and exploration time of the two objects) or an unpaired *t*-test (LMA and DI) were used to analyse the NOR test data. All statistical analysis was performed using GraphPad Prism 9.3.1. Statistical significance was set at *p* < 0.05. Averaged values are expressed as mean ± SEM.

#### 2.7.2. Electrophysiological Data

**Figure 1:**
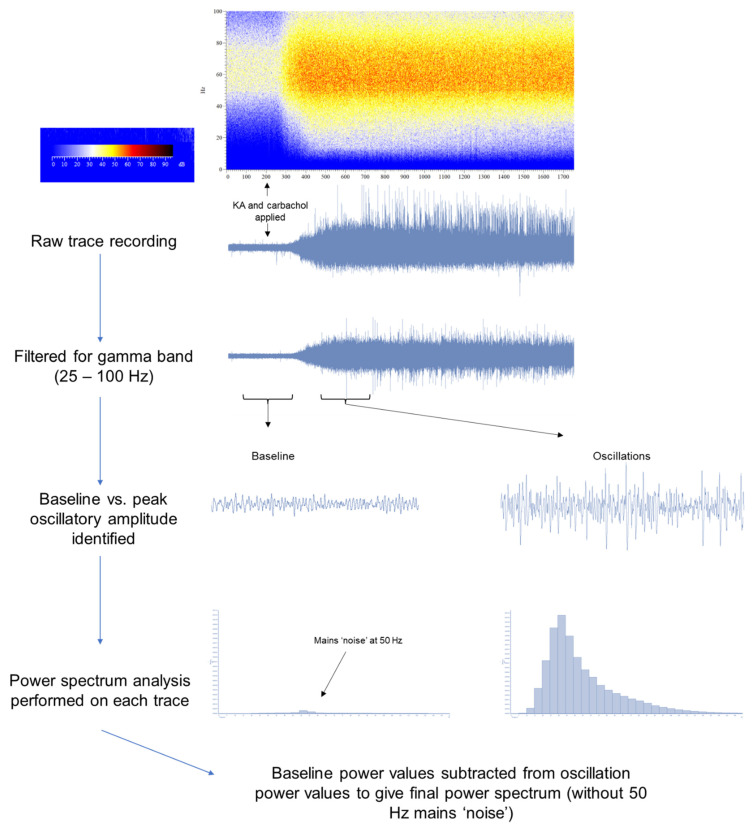
The analysis protocol used to acquire oscillatory power values from each slice recording. The raw trace recording from each PFC slice (illustrated by the example spectrogram and waveform) was filtered for the gamma (25–100 Hz) band. From the filtered trace, 100–300 s sections of the baseline and peak oscillatory amplitude were identified. After a power spectrum analysis was performed on the baseline trace and oscillation trace, the baseline power values were subtracted from the oscillation values to remove any mains ‘noise’ present at 50 Hz. The final power spectrum values were then produced from this slice and used to calculate amplitude and area power for each recording.

KA and carbachol-elicited gamma oscillations were characterised using power spectral density (PSD) analysis. PSD profiles of the field potential recordings from the PFC were generated by Fourier transform analysis using Spike2 (CED) software (Hanning window, FFT size 2048, resolution 4.883 Hz). PSD profiles were calculated from a 100–300 s epoch of the field potential trace displaying peak power amplitude, filtered for the gamma frequency band (25–100 Hz) and with the baseline power subtracted offline to remove the noise of the 50Hz mains (illustrated in Figure 1). Area power was also calculated for each recording as the area under the peak in each power spectrum. Recordings were conducted on three slices from each animal in a random order (to prevent any time effects). An animal was only included in the analysis if robust oscillations were yielded from all three slices: thus, a total of five animals across all Cohorts were excluded. Recordings for Cohort 1 were performed on one control slice, one treated with VU0409551 and the other with VU0360172. Within each group of animals (scVehicle, *n* = 30 slices; scPCP, *n* = 30 slices), treatment effects (control, *n* = 10; VU0409551 5 µM, *n* = 10; VU0360172 5 µM, *n* = 10) in amplitude power were compared using a 2-way ANOVA, matched by animal and frequency (Hz), and differences in area power were compared using a Friedman’s analysis followed by a Dunn’s multiple comparisons tests. The amplitude and area power of control slices (scVehicle, *n* = 10 slices; scPCP, *n* = 10 slices) was also compared between scVehicle and scPCP animals, using, respectively, a 2-way ANOVA matched by frequency and a Mann-Whitney test. For experiments in Cohort 2 testing PAMs at the lower concentration of 2 µM (*n* = 5 per group) and those using mGlu5 receptor, PKC, and PLC inhibitors (all *n* = 4 per group), differences in amplitude and area power between treatment groups were compared using a Friedman’s analysis followed by a Dunn’s multiple comparisons tests. All statistical analysis was performed using GraphPad Prism 9.3.1. Differences were reported as statistically significant at *p* < 0.05. Averaged values are expressed as mean ± SEM.

## 3. Results

### 3.1. Novel Object Recognition (NOR) Is Impaired in the scPCP Model

**Figure 2:**
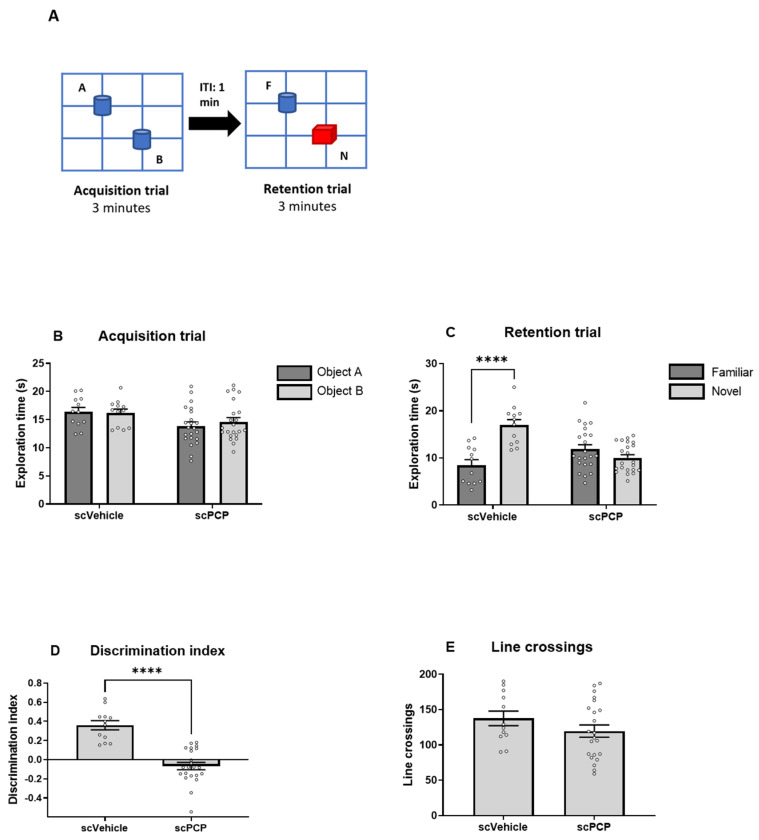
Verification that sub-chronic PCP (scPCP) treatment impairs novel object recognition. (**A**) Graphic depicting the novel object recognition (NOR) task protocol. A 3 min acquisition trial (with two identical objects, A and B in blue) is followed by a 1 min inter-trial interval (ITI) before a 3 min retention trial (with familiar object, F blue and novel object, N red). (**B**,**C**) The impact of scPCP treatment (2 mg/kg, i.p. twice daily for seven days, followed by a 6-week washout period) on the exploration time (s) of two identical objects in the acquisition phase and a familiar versus novel object in the retention phase. Data are expressed as mean ± S.E.M (*n* = 12–22 per group) and were analysed using a 2-way ANOVA. **** *p* < 0.0001; Significant increase in exploration time of the novel relative to the familiar object. (**D**) The effect of scPCP treatment on the discrimination index (DI). Data are presented as the mean ± S.E.M (*n* = 12–22 per group) and were analysed using an unpaired *t*-test. **** *p* < 0.0001; Significant reduction in DI relative to scVehicle. (**E**) The effect of scPCP treatment on total number of line crossings in the acquisition and retention trials. Data are presented as the mean ± S.E.M (*n* = 12–22 per group) and were analysed using an unpaired *t*-test.

The NOR paradigm (Figure 2A), performed following a 6-week washout period, was used to assess scPCP-induced visual recognition memory deficits in animals which were then subsequently used for the electrophysiological recording of oscillations. A 2-way ANOVA found no significant interaction between scPCP/scVehicle treatment and object exploration during the acquisition trial, with no significant difference in the exploration time of two identical objects (A and B) for either treatment group (Figure 2B). However, in the retention trial, there was a significant interaction between scPCP/scVehicle treatment and object exploration as revealed by a 2-way ANOVA (F (1, 32) = 54.48; *p* < 0.0001). As anticipated, scVehicle rats showed a significant preference for exploring the novel (N) relative to the familiar (F) object (t (22) = 5.147; *p* = 0.000037), whilst similar exploration times in scPCP rats demonstrated an impaired ability to differentiate between the novel and familiar objects (Figure 2C). This was highlighted by the significantly lower discrimination index in the scPCP rats compared with scVehicle rats (t (32) = 6.798; *p* < 0.000001; Figure 2D). However, no significant difference in locomotor activity (the total number of line crossings across both trials) was found between the scVehicle and scPCP treatment groups (Figure 2E). Thus, as previously reported, the scPCP model exhibits specific disruption of NOR performance without locomotor deficits [36].

### 3.2. scPCP Treatment Is Associated with Diminished Gamma Oscillations

**Figure 3:**
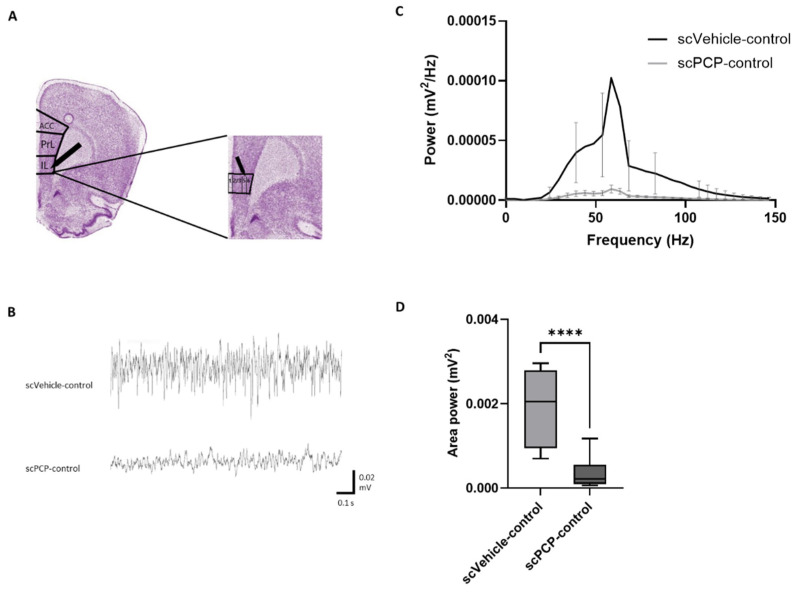
scPCP treatment significantly reduces gamma oscillatory power. (**A**) Graphic illustrating the location of the recording electrode in prefrontal cortex (PFC) slices: layer V of the infralimbic cortex (IL). Other regions indicated are the anterior cingulate cortex (ACC) and prelimbic cortex (PrL). Image adapted from the rat brain atlas [53]. (**B**) Representative traces showing oscillatory activity in PFC slices from scPCP-treated and scVehicle-treated rats. (**C**) Power spectrum analysis displaying amplitude power for scPCP vs. scVehicle slices (*n* = 10 per group, analysed using a 2-way ANOVA matched by frequency). (**D**) Box plot showing the median (whiskers max–min) area power in slices from scPCP and scVehicle rats (*n* = 10 per group, analysed using Mann-Whitney test; **** *p* < 0.0001, significant decrease in area power compared to scVehicle group).

Control slices from scVehicle and scPCP rats in Cohort 1 were incubated with aCSF for 1 h prior to the bath application of KA and carbachol. Extracellular field potential activity was recorded using a microelectrode filled with aCSF positioned in layer V of the infralimbic (IL) cortex (Figure 3A). Oscillatory power was significantly diminished in PFC slices from scPCP rats relative to scVehicle rats (representative traces in Figure 3B). For amplitude power, a 2-way ANOVA test demonstrated a significant interaction between frequency and treatment (scVehicle vs. scPCP; Figure 3C; F (30, 540) = 2.119; *p* = 0.0006). For area power, a Mann-Whitney test showed a significant reduction in scPCP relative to scVehicle slices (Figure 3D; U = 3; *p* < 0.0001). These results demonstrate that the effects of scPCP treatment on the PFC results in a significant reduction in oscillatory power.

### 3.3. scPCP-Induced Impairments in Gamma Oscillations Are Restored by VU0409551 and VU0360172 in a Concentration-Dependent Manner

**Figure 4:**
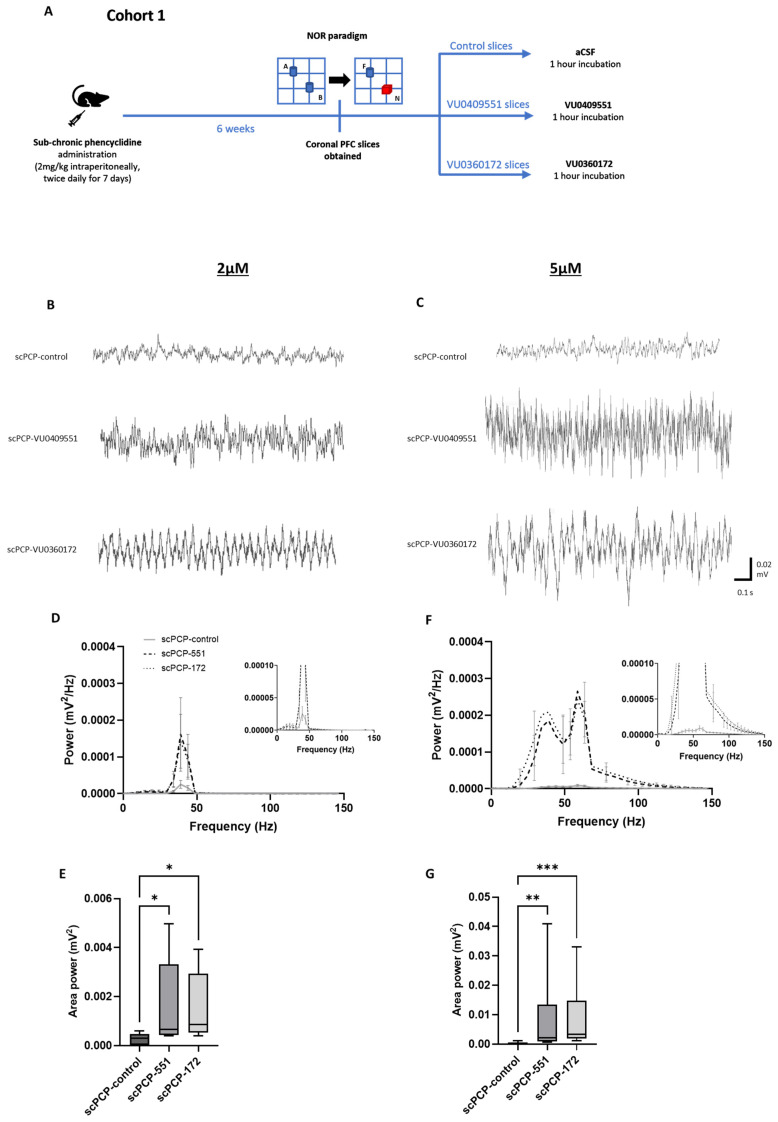
Incubation of scPCP slices with either VU0409551 or VU0360172 significantly increased gamma oscillatory power. (**A**) Schematic illustrating the experimental protocol for Cohort 1. A, B, blue; identical objects. F, blue; familiar object. N, red; novel object. (**B**,**C**) Representative traces showing oscillatory activity in PFC slices from scPCP-treated rats without PAM application (scPCP-control), and scPCP slices treated with VU0409551 or VU0360172. Power spectrum analyses show the effect of VU0409551 or VU0360172 incubation at (**D**), 2 µM (*n* = 5 per group, analysed using a Friedman’s test followed by Dunn’s tests) and (**F**), 5 µM (*n* = 10 per group, analysed using a 2-way ANOVA) on amplitude power. Graph insets more clearly illustrate data in low power ranges. Box plots show the median (whiskers max–min) area power in slices incubated in VU0409551 or VU0360172 at (**E**), 2 µM (*n* = 5 per group) and (**G**), 5 µM (*n* = 10 per group; both analysed using Friedman’s tests followed by Dunn’s tests; * *p* < 0.05, ** *p* < 0.01, *** *p* < 0.001, significant increase in area power compared to scPCP-control group).

As summarised in Figure 4A, additional slices from scVehicle and scPCP rats in Cohort 1 were incubated with the mGlu5 receptor PAMs either VU0409551 or VU0360172 (2–5 µM) for 1 h prior to the bath application of KA and carbachol. Oscillatory power was significantly increased in PFC slices from scPCP rats incubated with either of the PAMs compared to scPCP-control slices (representative traces in Figure 4B,C).

A Friedman’s analysis showed that there was a significant difference in amplitude power between scPCP slices in control, VU0409551 and VU0360172 treatment conditions at a concentration of 2 µM (Figure 4D; Fr = 55.03, *p* < 0.0001). Dunn’s multiple comparisons tests revealed scPCP slices to show a significant increase in amplitude power following incubation with 2 µM VU0409551 or VU0360172 relative to control slices (both *p* < 0.0001). Furthermore, Friedman’s analysis demonstrated a significant difference in area power between the 3 treatment conditions (Figure 4E; Fr = 7.6, *p* = 0.0239), with Dunn’s tests showing a significant increase in area power following incubation with 2 µM VU0409551 (*p* = 0.0114) or VU0360172 (*p* = 0.0269) relative to control slices.

At a concentration of 5 µM, 2-way ANOVA tests revealed a significant interaction between frequency and treatment (control, VU0409551, VU0360172) for amplitude power (Figure 4F; F (30, 270) = 7.252; *p* < 0.0001). A significant increase in amplitude power relative to control slices was induced by 5 µM VU0409551 incubation (F (30, 270) = 3.127, *p* < 0.0001) and 5 µM VU0360172 incubation (F (30, 270) = 5.745, *p* < 0.0001). Furthermore, Friedman’s analysis demonstrated a significant difference in area power between the 3 treatment conditions (Figure 4G; Fr = 18.27, *p* = 0.0001), with Dunn’s tests showing a significant increase in area power following incubation with 5 µM VU0409551 (*p* = 0.0026) or VU0360172 (*p* = 0.0002) relative to control slices.

The smaller increase in power relative to the control induced by the lower concentration (of 2 µM) indicates a concentration-dependent relationship in the effects of the PAMs on oscillatory activity, and 5 µM was selected as the most effective concentration to employ in subsequent experiments. There was no significant increase in oscillatory power in PFC slices from scVehicle rats incubated with either PAM compared to scVehicle-control slices (Figure S1, Supplementary Material). To exclude the possibility that the PAMs themselves induced oscillations during the incubation period and this somehow affected the subsequent oscillations induced by KA and carbachol-, we measured oscillatory activity during PAM incubation. No oscillations were induced during PAM incubation for any of the slices, with the baseline power not significantly different from what was measured in the control slices (incubated in aCSF). These results imply that incubation with either VU0409551 or VU0360172 reversed the scPCP-induced deficits in oscillatory power, in a concentration-dependent manner, with VU0409551 incubation inducing a greater increase in oscillations relative to control than VU0360172.

### 3.4. The Effect of mGlu5 Antagonism on the PAM-Mediated Enhancement of scPCP Gamma Oscillations

**Figure 5:**
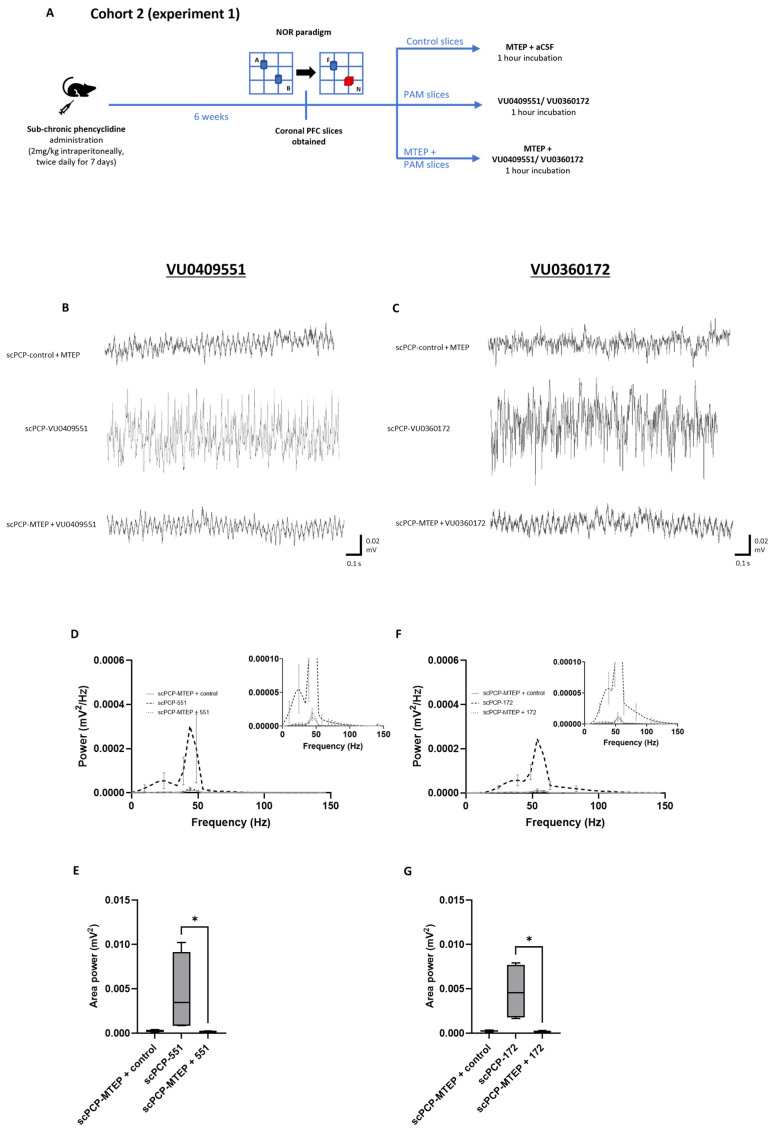
The mGlu5 receptor antagonist, MTEP prevents the restorative effects of VU0409551 or VU0360172 on scPCP slices in terms of gamma power. (**A**) Schematic illustrating the experimental protocol for Cohort 2, experiment 1. A, B, blue; identical objects. F, blue; familiar object. N, red; novel object. (**B**,**C**) Representative traces showing oscillatory activity in PFC slices from scPCP-treated rats without PAM application with MTEP alone (scPCP-control + MTEP), and scPCP slices treated with MTEP followed by VU0409551 or VU0360172. The MTEP was present during PAM incubation but was washed off before oscillations were evoked. Power spectral analyses show the effect of MTEP alongside (**D**), VU0409551, and (**F**), VU0360172 on amplitude power (*n* = 4 per group, analysed using a Friedman’s test followed by Dunn’s tests). Graph insets more clearly illustrate data in low power ranges. Box plots show the median (whiskers max–min) area power in slices incubated in (**E**), VU0409551 (*n* = 4 per group) or (**G**), VU0360172 (*n* = 4 per group; both analysed using Friedman’s tests followed by Dunn’s tests; * *p* < 0.05, significant decrease in area power compared to PAM incubation alone).

To verify that the mGlu5 receptor PAM-mediated restoration of functional oscillatory activity in scPCP animals was due to modulation of the mGlu5 receptor, in Cohort 2 we tested the effect of co-incubating PFC slices with each PAM together with the mGlu5 receptor antagonist MTEP (applied 20 min prior to the PAM incubation) in comparison to slices treated with the PAM alone and control slices (co-incubated with aCSF + MTEP), as summarised in Figure 5A. Oscillatory power was significantly increased in PFC slices from scPCP rats incubated with either PAM compared to those co-incubated with MTEP and a PAM or scPCP-control slices (representative traces in Figure 5B,C).

There was a significant difference between the control, VU0409551, and MTEP + VU0409551 slices in terms of amplitude power (Figure 5D; Fr = 50.13, *p* < 0.0001). Dunn’s multiple comparisons tests revealed that scPCP slices incubated with MTEP + VU0409551 showed no significant difference in amplitude power compared to control slices, and a significant decrease in amplitude power relative to incubation with VU0409551 alone (*p* < 0.0001). Moreover, there was a significant difference in area power between the 3 treatment conditions (Figure 5E; Fr = 6.5, *p* = 0.0417), with Dunn’s tests showing a significantly greater area power in slices incubated with VU0409551 relative to MTEP + VU0409551 (*p* = 0.04).

There was also a significant difference between the control, VU0360172, and MTEP + VU0360172 slices in amplitude power (Figure 5F; Fr = 62.0, *p* < 0.0001). Again, Dunn’s multiple comparisons tests revealed that scPCP slices incubated with MTEP + VU0360172 showed no significant difference in amplitude power compared to control slices, and a significant decrease in amplitude power relative to incubation with VU0360172 alone (*p* < 0.0001). Moreover, there was a significant difference in area power between the 3 treatment conditions (Figure 5G; Fr = 6.5, *p* = 0.0417), with Dunn’s tests showing a significantly greater area power in slices incubated with VU0409551 relative to MTEP + VU0409551 (*p* = 0.04).

Taken together, these data imply that the capacity of the PAMs to enhance gamma power in scPCP slices is due to their specific modulation of the mGlu5 receptor.

### 3.5. The Effect of PKC Inhibition on the PAM-Mediated Enhancement of scPCP Gamma Oscillations

**Figure 6:**
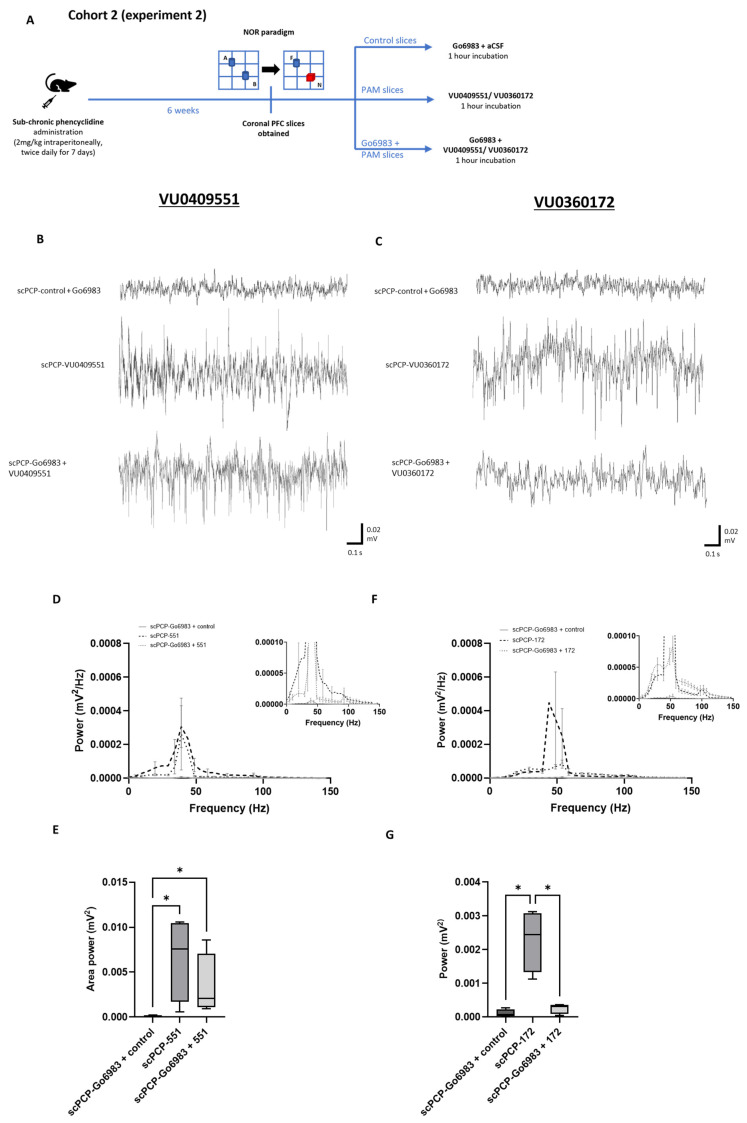
Go6983 (10 nM) had differential effects on the activity of VU0409551 or VU0360172 in scPCP slices in terms of gamma power. (**A**) Schematic illustrating the experimental protocol for Cohort 2, experiment 2. A, B, blue; identical objects. F, blue; familiar object. N, red; novel object. (**B**,**C**) Representative traces showing oscillatory activity in PFC slices from scPCP-treated rats without PAM application with Go6983 alone (scPCP-control + Go6983), and scPCP slices treated with Go6983 followed by VU0409551 or VU0360172. The Go6983 was present during PAM incubation but was washed off before oscillations were evoked. Power spectral analyses show the effect of Go6983 alongside (**D**), VU0409551, and (**F**), VU0360172 on amplitude power (*n* = 4 per group, analysed using a Friedman’s test followed by Dunn’s tests). Graph insets more clearly illustrate data in low power ranges. Box plots show the median (whiskers max–min) area power in slices incubated in (**E**), VU0409551 (*n* = 4 per group) or (**G**), VU0360172 (*n* = 4 per group; both analysed using Friedman’s tests followed by Dunn’s tests; * *p* < 0.05, significant increase in area power in with PAM incubation or Go6983 + VU0409551 incubation relative to control; * *p* < 0.05, significant decrease in area power following Go6983 + VU0360172 incubation relative to VU0360172 alone).

To investigate the phenomenon of ‘modulation bias’ in the action of these mGlu5 receptor PAMs, we co-incubated scPCP slices from Cohort 2 with each PAM alongside the PKC inhibitor Go6983 (10 nM applied 20 min prior to the PAM), as summarised in Figure 6A. Oscillatory power was then compared with activity from control slices (co-incubated with aCSF + Go6983) and slices treated with the PAM alone (representative traces in Figure 6B,C).

There was a significant difference between the control, VU0409551, and Go6983 + VU0409551 slices in terms of amplitude power (Figure 6D; Fr = 58.26, *p* < 0.0001). Dunn’s multiple comparison tests revealed that PFC slices from scPCP rats incubated with Go6983 + VU0409551 showed significantly greater amplitude power compared to scPCP-control slices (*p* < 0.0001). Go6983 + VU0409551 slices showed a small reduction in power relative to incubation with VU0409551 alone, but this was not statistically significant. Moreover, there was a significant difference in area power between the 3 treatment conditions (Figure 6E; Fr = 6, *p* = 0.0394), with Dunn’s tests showing significantly greater area power in slices incubated with VU0409551 or Go6983 + VU0409551 relative to scPCP-control slices (both *p* = 0.0339) but no significant difference between area power in VU0409551 or Go6983 + VU0409551 slices.

There was also a significant difference between the control, VU0360172, and Go6983 + VU0360172 slices in terms of amplitude power (Figure 6F; Fr = 55.03, *p* < 0.0001). In contrast to VU0409551, scPCP slices incubated with Go6983 + VU0360172 showed a significant reduction in amplitude power compared to incubation in VU0360172 alone (*p* = 0.0105). Go6983 + VU0360172 treatment was, however, associated with greater amplitude power relative to the control (*p* < 0.0001). There was a significant difference in area power between the 3 treatment conditions (Figure 6G; Fr = 6, *p* = 0.0394), with Dunn’s tests showing a significantly greater area power in slices incubated with VU0360172 relative to Go6983 + VU0360172 or control slices (both *p* = 0.0339).

Blocking the PKC pathway did not significantly affect the activity of VU0409551 but significantly reduced the activity of VU0360172, indicating a possible ‘bias’ in the activity of VU0409551 away from the PKC/MAPK signalling pathway and potentially toward the PI3K/Akt pathway.

### 3.6. The Effect of PLC Inhibition on the PAM-Mediated Enhancement of scPCP Gamma Oscillations

**Figure 7:**
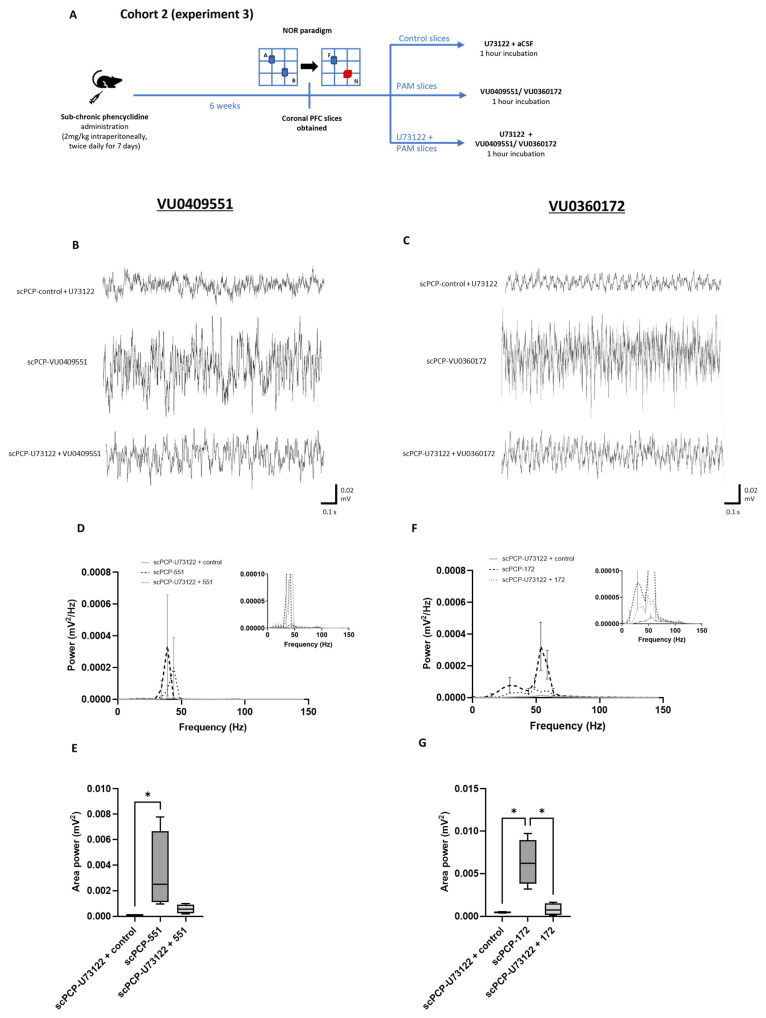
U73122 (10 µM) reduced the activity of VU0409551 or VU0360172 in scPCP slices in terms of gamma power. (**A**) Schematic illustrating the experimental protocol for Cohort 2, experiment 3. A, B, blue; identical objects. F, blue; familiar object. N, red; novel object. (**B**,**C**) Representative traces showing oscillatory activity in PFC slices from scPCP-treated rats without PAM application with U73122 alone (scPCP-control + U73122), and scPCP slices treated with U73122 followed by VU0409551 or VU0360172. The U73122 was present during PAM incubation but was washed off before oscillations were evoked. Power spectral analyses show the effect of U73122 alongside (**D**) VU0409551, and (**F**), VU0360172 on amplitude power (*n* = 4 per group, analysed using a Friedman’s test followed by Dunn’s tests). Graph insets more clearly illustrate data in low power ranges. Box plots show the median (whiskers max–min) area power in slices incubated in (**E**), VU0409551 (*n* = 4 per group) or (**G**), VU0360172 (*n* = 4 per group; both analysed using Friedman’s tests followed by Dunn’s tests; * *p* < 0.05, significant increase in area power in with PAM incubation relative to control; * *p* < 0.05, significant decrease in area power following U73122 + VU0360172 incubation relative to VU0360172 alone).

Since PLC is upstream of both PKC activation and intracellular calcium mobilisation and downstream of mGlu5 receptor-mediated Gαq/11 activation, PLC blockade was used to investigate the nature of PAM-induced intracellular signalling mechanisms. If intracellular calcium release is important for the activity of both PAMs, then blocking PLC should reduce their effect on oscillatory activity. As summarised in Figure 7A, we applied each PAM to scPCP slices from Cohort 2 in combination with the PLC inhibitor U73122 (10 µM administered 20 min prior to the PAM) and compared oscillations with activity from control slices (co-incubated with aCSF + U73122) and slices incubated with the PAM alone (representative traces in Figure 7B,C).

There was a significant difference between the control, VU0409551, and U73122 + VU0409551 slices in terms of amplitude power (Figure 7D; Fr = 29.87, *p* < 0.0001). Dunn’s multiple comparison tests revealed that scPCP slices incubated with U73122 + VU0409551 showed a significant decrease in amplitude power relative to incubation with VU0409551 alone (*p* = 0.0105). However, U73122 + VU0409551 slices also showed significantly greater amplitude power relative to control slices (*p* < 0.0001). Additionally, there was a significant difference in area power between the 3 treatment conditions (Figure 7E; Fr = 8.3, *p* = 0.0046); Dunn’s tests showed a significant increase in area power in slices incubated with VU0409551 relative to control slices (*p* = 0.0339), and there was a non-significant reduction in area power in U73122 + VU0409551 slices relative to VU0409551 alone.

Similarly, there was a significant difference between the control, VU0360172, and U73122 + VU0360172 slices in terms of amplitude power (Figure 7F; Fr = 58.06, *p* < 0.0001). Dunn’s multiple comparison tests revealed that scPCP slices incubated with U73122 and VU0360172 showed a significant decrease in amplitude power relative to incubation with VU0360172 alone (*p* = 0.0004). However, U73122 and VU0409551 incubated slices also showed significantly greater amplitude power relative to control slices (*p* = 0.0004). Finally, there was a significant difference in area power between the 3 treatment conditions (Figure 7G; Fr = 6, *p* = 0.0494), with Dunn’s tests showing a significantly greater area power in slices incubated with VU0360172 relative to U73122 + VU0360172 or control slices (both *p* = 0.0339).

This suggests that blocking intracellular calcium release in addition to downstream PKC signalling reduced (but did not eliminate) the effects of both VU0409551 and VU0360172, implying that intracellular calcium mobilisation is probably involved in the activity of both PAMs, but that other pathways are also activated.

## 4. Discussion

In this study, we have shown for the first time that two mGlu5 receptor PAMs, VU0409551 and VU0360172, are efficacious in restoring gamma oscillations in PFC slices from the scPCP rodent model of CIAS. We have also explored the mechanism of action of these PAMs, verifying the requirement of mGlu5 receptor activation and investigating the phenomenon of ‘modulation bias’. Both PAMs appear to induce PLC activation, with VU0409551 exhibiting a reduced preference for activating the PKC cascade and VU0360172 stimulating both PKC and PI3K intracellular signalling pathways. These data suggest that an increase in gamma power could be one of the underlying mechanisms for how these PAMs improve cognitive function in this model. Further investigation is warranted into ‘modulation bias’ and its consequences, particularly pertaining to VU0409551 and the possibility of developing new derivatives.

As anticipated from previous research, scPCP animals displayed significantly impaired NOR performance compared to scVehicle rats [36,54]. As reviewed by Neill et al. [55], the NOR paradigm assesses visual recognition memory, one of the seven cognitive domains fundamentally impaired in schizophrenia as identified by the Measurement and Treatment Research to Improve Cognition in Schizophrenia (MATRICS) initiative. The exclusive use of female animals in the scPCP model is because, in comparison to males, female rats are evidenced to perform better in cognitive tasks and are more susceptible to the effects of PCP [51,56,57]. It should be noted that the oestrous cycle was not assessed in this study, meaning NOR performance may have been influenced by female rats being in distinct phases of oestrous during the task. Whilst some literature suggests improved object recognition in Long-Evans rats during the oestrogen peak at pro-oestrous [58], the NOR performance of Lister Hooded rats was unaffected by the oestrous cycle stage [56]. In addition, the stress induced by oestrous cycle monitoring has been shown to alter female rodent behaviour [59,60]. Animals showing a significantly greater exploration of the novel object in the retention phase reflect the functional recognition memory steps of storage, consolidation, and retrieval [61]. Conversely, animals with cognitive impairment are expected to show comparable exploration times of both novel and familiar objects [62]. The significantly lower discrimination index of scPCP relative to scVehicle rats thereby validates scPCP treatment as a model for CIAS in the current study.

**Figure 8:**
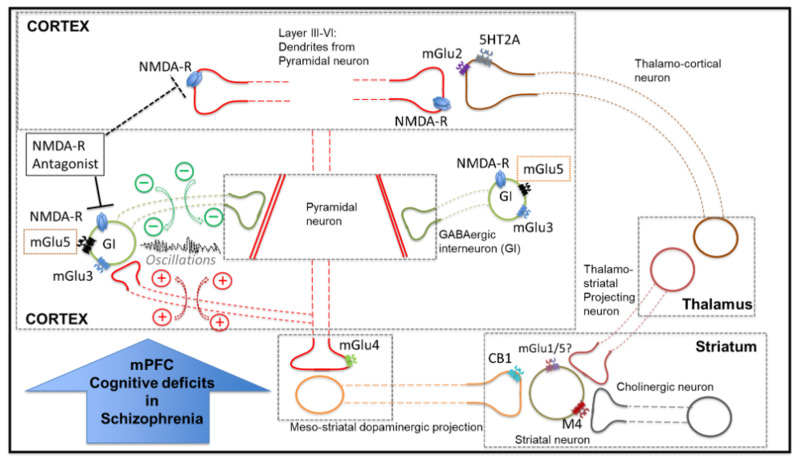
Diagram illustrating an overview of mGlu receptor subtypes and other relevant receptors involved in a mechanistic network in the pathophysiology of schizophrenia. A hypothetical role of the mGlu5 receptor (mGlu5) in this network involves connectivity between an excitatory pyramidal neuron (in red) and inhibitory interneurons expressing mGlu5 (in green). The inhibitory interneurons, including parvalbumin-expressing neurons, are dynamically positioned to produce synchronous oscillations, such as gamma-band oscillations, through recurrent feedback inhibition (GABAergic interneurons in green) and excitation by pyramidal neurons (collateral axon projection indicated in red). This connectivity is disturbed in schizophrenia, disrupting gamma oscillations, and producing cognitive deficits. mGlu5 receptor subtypes are expressed in interneurons (in green) and in pyramidal neurons (not shown here). In addition, functional interaction between mGlu3 and mGlu5 has been demonstrated in the CNS, with mGlu3 receptor activation enhancing mGlu5 signalling [80]. Speculatively, group I mGluR (mGlu1/5) activation also induces the release of endocannabinoids, which in turn activate presynaptic CB1 receptors on mesostriatal dopaminergic neurons, thereby reducing dopamine release. Finally, mGlu5 receptor subtypes are functionally coupled with N-methyl-D-aspartate (NMDA) receptors. Biased pure mGlu5 receptor PAMs that do not induce NMDA receptor activation are devoid of toxic effects related to NMDA coupling. Derivatives of these PAMs such as VU0409551 and VU0360172 that do not induce coupling of mGlu5 receptors to NMDA receptors are under development for the unmet therapeutic need of CIAS.

Here, extracellular field potential activity was recorded in layer V of the infralimbic (IL) cortex (Figure 3A). This was to assess changes in the PFC microcircuit associated with schizophrenia, as illustrated in Figure 8. This network is composed of PV basket interneurons which deliver GABAergic inhibitory signals to the perisomatic region of pyramidal cells, which in turn mediate glutamatergic excitation [14,17]. PV GABAergic neurons constitute the largest sub-class of layer V interneurons, are heavily involved in producing synchronised oscillations, and are evidenced to suffer alterations in schizophrenia [18,19,20,63,64,65,66,67,68,69,70]. Carbachol and kainate were used in light of existing literature demonstrating the ability of these compounds to elicit stable persistent fast gamma oscillations across layers III-VI in each subregion of the medial PFC (prelimbic, infralimbic, and dorsopeduncular) in an interface-style chamber [71]. Although we have observed significant effects on the onset of oscillations, we cannot exclude the possibility that with longer duration recordings of the oscillations (2–3 h) the observed effects of the experimental conditions may be different. However, these 30 min recordings allowed data acquisition from the maximal number of slices per animal and provided insight into the differences in gamma power shown in the initial oscillatory onset period. In these experimental conditions, scPCP treatment was not only associated with deficits in visual recognition memory, but also with reduced gamma oscillatory power recorded in the IL cortex of ex vivo PFC slices.

Mounting evidence has implicated disturbances in neural synchrony and gamma oscillations as major pathophysiological features underlying the core cognitive dysfunctions of schizophrenia. Some authors have hypothesised that the progressive oscillatory deficits of schizophrenia reflect the abnormal development of cortical inhibitory circuits [72]. Indeed, the functional maturation of the PFC is not completed until late adolescence and early adulthood [73,74,75]. This period of development is not only when the symptoms of schizophrenia usually manifest, but it is also when PV neuronal circuits reach maturation [76,77] and gamma oscillatory power increases [78,79].

As the PFC intrinsically oscillates at high frequencies, during which GABAergic deficits also become functionally apparent, gamma oscillations have been widely investigated in relation to schizophrenia [81]. Preclinically, chronic ketamine treatment has been shown to reduce the peak oscillatory frequency of KA-induced oscillations in mouse PFC slices, with the power of 40–50 Hz gamma oscillations particularly diminished [22]. The NMDAR antagonists ketamine and MK-801 have also been shown to produce sensory-evoked gamma deficits accompanied by disrupted prepulse inhibition (PPI). Interestingly, clozapine was the only antipsychotic drug tested that restored gamma oscillations and attenuated the disruption to PPI [82]. These findings are paralleled in clinical studies, with gamma-band EEG-evoked responses to direct frontal cortex stimulation decreased in schizophrenia patients [23] and reduced perceptual organization task performance by individuals with schizophrenia accompanied by a diminished power of 60–120 Hz gamma oscillations [83]. The rise in gamma power with working memory load observed in healthy individuals is also absent in schizophrenia patients [84,85].

Here, we show that two distinct mGlu5 receptor PAMs significantly increase gamma power in slices from scPCP rats in a concentration-dependent manner. Interestingly, the PAMs have no effect on gamma power in slices from scVehicle rats (Figure S1, Supplementary Material) and thus the effects are selective for when the cortical network is dysfunctional. mGlu5 receptor blockade using MTEP inhibited this increased amplitude, confirming that PAM-mediated restoration of functional oscillatory activity in scPCP animals is due to modulation of the mGlu5 receptor. The contribution of the mGlu5 receptor to oscillatory activity is supported by many previous studies. Inhibition of the mGlu5 receptor in rats using MPEP has been shown to diminish gamma oscillations in the dentate gyrus whilst impairing spatial memory [86]. mGlu5 receptor knockout mice also display abnormalities in oscillatory activity associated with schizophrenia, and specific deletion of the mGlu5 receptor from PV interneurons reduced PV density and inhibitory signals and modified oscillations [87,88]. Allosteric potentiation of mGlu5 by ADX47273 also induces a more significant elevation in gamma oscillatory power in rats than in MPEP or control treatment [89]. Taken alongside our previous work demonstrating the in vivo efficacy of VU0409551 and VU0360172 in reversing the scPCP-induced cognitive deficit, this evidence suggests that mGlu5 receptor PAMs are a promising strategy for correcting the abnormalities in gamma oscillations and cognition seen in schizophrenia [36].

The fact that incubation of PFC slices for 1 h with the PAMs restores oscillatory activity suggests that firstly, the PAMs act directly in the PFC with no requirement for downstream or upstream structures. Secondly, there must be enough network activity in the PFC slices to induce mGlu5 receptor activation following PAM application and 1 h is sufficient to induce lasting effects on oscillations. These effects are hypothesised to represent changes in protein expression or location. A recent study [90] showed that incubation of thalamic slices in VU030172 (5 μM) for 1 h could change the tonic GABA-A receptor current, an effect that appeared to result from a change in GABA uptake and GAT-1 expression. These effects occurred without changes in GAT-1 mRNA and thus do not represent increased transcription but could result from GAT-1 trafficking or increased translation. Such alterations could underlie the increased oscillatory power observed in the current study.

**Figure 9:**
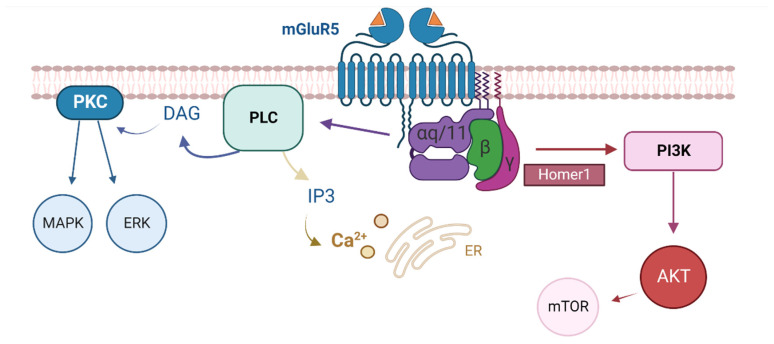
Graphic illustrating the intracellular signalling pathways downstream of mGlu5 receptor activation. mGlu5 receptor-Gαq/11 coupling stimulates phospholipase C (PLC) to generate inositol-1,4,5-triphosphate (IP3), resulting in intracellular calcium mobilisation. Activated PLC also produces DAG, which phosphorylates PKC, MAPK, and ERK and impacts ion channel signalling. mGlu5 receptor stimulation can alternatively stimulate the phosphatidyl-inositol-3kinase (PI3K) pathway, phosphorylating AKT and activating the mammalian target of rapamycin (mTOR). Created with BioRender.com, accessed on 27 May 2022.

As illustrated in Figure 9, activation of the mGlu5 receptor induces signalling via multiple independent intracellular pathways. The ability of G-protein-coupled receptors (GPCRs) to form distinct ligand-induced configurations means that agonists may not equally activate all such intracellular signalling pathways downstream of the receptor [91]. Further complexity is introduced by the coupling of an allosteric modulator to an agonist-bound receptor, which can induce receptor activation states and functional outcomes that cannot be attained by the endogenous ligand alone [92]. Differential activation of these pathways by different ligands has been previously observed in group 1 mGlu receptors and the therapeutic consequences of this must be considered [93,94]. mGlu5 receptor signalling bias has also been linked to particular cognitive effects, with spontaneous memory loss associated with PFC mGlu5 receptor signalling bias towards a PI-independent pathway [95]. Here, we show that the effects of both VU0409551 and VU0360172 are at least partly mediated by PLC activation, which induces downstream calcium mobilisation. Through the coupling of calcium and calmodulin and activation of calcium/calmodulin-dependent kinases (CaMK), effects involved in the molecular mechanisms of memory and learning such as LTP are induced [42,96]. However, even with PLC inhibition, gamma power was still significantly elevated relative to control slices, suggesting that alternative pathways are also involved in the effects of both PAMs. VU0409551 appears to show a reduced preference for activation of the PKC cascade relative to VU0360173, and, as such, may be ‘biased’ toward the PI3K pathway, which leads to the phosphorylation of Akt. If the mGlu5 receptor preferentially activates select signalling pathways to the exclusion of others upon binding VU0409551, this could translate to favourable induction of certain physiological responses and behavioural outcomes. This may explain the finding that VU0409551 incubation of scPCP slices in this study induced a greater increase in oscillatory power relative to control than VU0360172. Biased allosteric modulators such as VU0409551 which activate only selective mGlu5 receptor pathways could thus be used to not only preclinically investigate the physiology of mGlu5 receptor-mediated beneficial versus adverse effects but also to mediate improved clinical therapeutic outcomes [92].

To conclude, the reduced amplitude and area of gamma oscillatory power in schizophrenia could be the result of various neuronal abnormalities, including reduced neuronal numbers, synaptic connectivity, and/or synchrony [97,98]. The reversal of scPCP-induced oscillatory deficits by mGlu5 receptor PAMs in this study provides support for the latter hypothesis. Diminished neuronal synchrony is suggested to reflect abnormalities in networks of GABA-ergic interneurons and/or disrupted glutamatergic signalling [99,100]. We hypothesise that mGlu5 receptor PAMs restore the excitatory/inhibitory signalling balance in cortical networks, thereby correcting neuronal synchrony and increasing oscillatory power in scPCP rats, which thus show improved cognitive performance. With mounting evidence for the ability of certain PAMs to activate intracellular signalling pathways, it is necessary to further assess the utility of this ‘modulation bias’ in targeting the mGlu5 receptor and consider how this could influence therapeutic outcomes. Only with such insight can effective pharmacological interventions for CIAS be developed and the functional recovery of patients significantly improved.

## Data Availability

The data presented in this study are contained within this article and Supplementary Materials.

## Supplementary Materials

The following supporting information can be downloaded at: https://www.mdpi.com/article/10.3390/cells12060919/s1, Figure S1: The mGlu5 receptor PAMs have little effect on oscillatory power in slices from scVehicle rats Power spectral analyses for (A), gamma-band (25–100 Hz) (B), beta-band (12.5–30 Hz) and C, theta-band (4–7 Hz) oscillations show the effect of VU0409551 or VU0360172 incubation of PFC slices from scVehicle animals on power (mV2; n = 10 per group, analysed using a 2-way ANOVA matched by animal and frequency).
