# Supplementary material for: Oscillatory Deficits in the Sub-Chronic PCP Rat Model for Schizophrenia Are Reversed by mGlu5 Receptor-Positive Allosteric Modulators VU0409551 and VU0360172
Source: Cells. 2023 Mar 16;12(6):919. doi: 10.3390/cells12060919 (PMC10047164; doi:10.3390/cells12060919)
Supplement: Supplementary file 1 [file cells-12-00919-s001.zip › cells-2237444-supplementary.pdf]

Supplementary Material

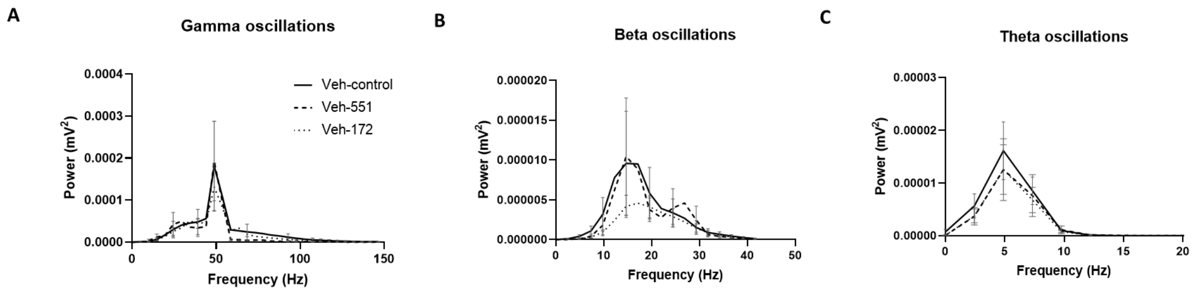

Figure S1 Supplementary Material. The mGlu5 receptor PAMs have little effect on oscillatory power in slices from scVehicle rats Power spectral analyses for **A,** gamma-band (25 – 100 Hz) **B,** beta-band (12.5 – 30 Hz) and C, theta-band (4 – 7 Hz) oscillations show the effect of VU0409551 or VU0360172 incubation of PFC slices from scVehicle animals on power (mV^2; n = 10 per group, analysed using a 2-way ANOVA matched by animal and frequency).
